# Peptides in Receptor-Mediated Radiotherapy: From Design to the Clinical Application in Cancers

**DOI:** 10.3389/fonc.2013.00247

**Published:** 2013-09-25

**Authors:** Catherine Lozza, Isabelle Navarro-Teulon, André Pèlegrin, Jean-Pierre Pouget, Eric Vivès

**Affiliations:** ^1^Institut de Recherche en Cancérologie de Montpellier, Montpellier, France; ^2^INSERM, U896, Montpellier, France; ^3^Université Montpellier 1, Montpellier, France; ^4^Institut Régional du Cancer Montpellier, Montpellier, France; ^5^Université Montpellier 2, Montpellier, France

**Keywords:** targeting-peptide, bifunctional chelator, radionuclides, cancer, radiotherapy

## Abstract

Short peptides can show high affinity for specific receptors overexpressed on tumor cells. Some of these are already used in cancerology as diagnostic tools and others are in clinical trials for therapeutic applications. Therefore, peptides exhibit great potential as a diagnostic tool but also as an alternative or an additional antitumoral approach upon the covalent attachment of a therapeutic moiety such as a radionuclide or a cytotoxic drug. The chemistry offers flexibility to graft onto the targeting-peptide either fluorine or iodine directly, or metallic radionuclides through appropriate chelating agent. Since short peptides are straightforward to synthesize, there is an opportunity to further improve existing peptides or to design new ones for clinical applications. However, several considerations have to be taken into account to optimize the recognition properties of the targeting-peptide to its receptor, to improve its stability in the biological fluids and its residence in the body, or to increase its overall therapeutic effect. In this review, we highlight the different aspects which need to be considered for the development of an efficient peptide receptor-mediated radionuclide therapy in different neoplasms.

## Introduction

Hundreds of receptors are expressed ubiquitously at the cell surface and some others are overexpressed only on specific cells depending on their function but also on their biological state. For instance during tumorigenesis of urogenital neoplasms, receptors such as somatostatin, bombesin, cholecystokinin, epidermal growth factor receptor (EGFR), human epidermal growth factor receptor 2 (HER2), several types of integrin receptors, or anti-Müllerian hormone receptor type II (AMHR-II), show a higher expression rate on cancer cells than on normal cells. Therefore they represent very attractive targets for concentrating radionuclides (or drugs) at the tumor sites either for diagnostic or therapeutic applications. Monoclonal antibodies (mAbs) were recognized to be very appropriate owing to the specific interaction with their ligand and became very popular as potential “magic bullets” to be used in cancer (1). Tumor targeting radiolabeled mAbs use *in vivo* dates from the 50s and 20 years later the first antibody-based clinical tumor localization was reported (2). They have later been approved for the localization and staging of colorectal, ovarian, breast, and prostate cancer while other antibodies (Zevalin^®^, Bexxar^®^) have been approved for the treatment of non-Hodgkin’s lymphoma. The concept of mAbs as targeted drug delivery systems can overcome many of the non-specific side effects associated with traditional cancer chemo- or radiotherapy. However, the cost of antibody-mediated immunodetection or immunotherapy remains rather high since their production requires specific and complex expression systems and their extraction, purification, and derivatization follow strict procedures and regulations to fit with the requirements of a subsequent therapeutic use in human. Alternatively several short peptides from 3 to 12 amino acids with appropriate affinity and specificity for various targeted receptors have been discovered over the last 40 years. Peptides present a molecular weight generally around 1500 Da, have cheap production costs, are quickly produced using automated synthesis, are not immunogenic, have normally deep solid tumor penetration, but also low bone marrow accumulation and relatively fast blood clearance (3).

The idea to use radiolabeled-peptides to target specific cells dates back to the 70s when a peptide with a good affinity for the melanotropin receptor was highly tritiated thus opening the way to therapeutic labeling with radionuclides. These peptides were later applied to *in vitro* and *in vivo* peptide receptor-mediated radiotherapy (4). However the results were disappointing because of a weak *in vivo* stability and a low rate of radiotoxicity at the tumor site to abolish tumor growth (3). In the early 80s, in depth structure-activity relationship studies performed on the somatostatin receptor led to the design of several reduced-size analogs which were the first peptides based radiopharmaceutical for tumor scintigraphy (5, 6). These peptides were also later used in radiotherapy upon grafting to a radionuclide (7). In this latter case, the use of small radiolabeled-peptides was indicated for patients with inoperable or metastasized tumors.

Analogs of amino acids can be then integrated instead of natural amino acids and then tested for their biological stability or their binding affinity to the receptor. Cyclization or multimerization of the targeting-peptide can be further evaluated, and the benefit of grafting various additional elements such as polyethylene glycol (PEG) to improve the overall pharmacokinetic properties of the targeting unit can be verified. Once the design of the targeting-peptide itself has been completed, the loading of the toxic moiety remains to be considered. This toxic moiety could be a cytotoxic drug or a radionuclide used either in a chemotherapy or in a radiotherapy context, respectively. The choice of the chelator, the spacer arm between the targeting-peptide, and the chelator and other modifications of the global targeting unit should be also evaluated since several reports suggested significant changes in the behavior of closely related targeting molecules (8–10). The final aim remains indeed to reach the tumor site with the best efficacy while strongly reducing the residence/accumulation in the other organs, including liver and kidneys. In this review, we highlight the main criteria susceptible to improve the design of targeting-peptides dedicated ultimately to induce the most efficient receptor-mediated radiotherapy in various neoplasms such as breast, ovarian, prostate, testicular, and urinary organs cancers as examples.

## Selection of Peptides for a Specific Receptor-Mediated Recognition

Most of the tumor targeting-peptides have been selected mainly by three methods [for a review: (11, 12)]. The first method consists in the identification of interacting peptide sequences from random phage display libraries. Basically, phages showing both good affinity and specificity for a given receptor are selected following successive rounds of selection. A sequencing step allows afterward the identification of the binding sequence, if any. One of the most known example reported 20 years ago and identifying this way is certainly the tripeptide RGD sequence which shows a peculiar affinity for the alpha-v beta-1 integrin receptor (13). Affinity of the RGD sequence for alpha-v beta-3 and alpha-v beta-5 integrin receptors has been also identified. The RGD sequence is present in various circulating proteins including fibronectin, vitronectin, osteopontin, collagen, thrombospondin, fibrinogen, and von Willebrand factor and all these circulating proteins bind individually at least one member of the integrin receptor family upon direct interactions with their commonly displayed RGD sequence (14). These interactions are highly selective for each circulating protein and each series of integrin heterodimers structured as two membrane-spanning subunits. A second method for selecting affinity peptides consists in the synthesis of one randomly made peptide on one individual bead of resin following a well-defined operating scheme based on a mixing procedure ensuring the generation of a peptides bank. This methodology has been named the “One Bead-One Compound” (OBOC) approach (15). Then, upon incubation of a tumor cell line with a wide set of beads, each harboring a unique peptide ligand, it could be possible to subsequently isolate and identify the beads coated by one or more layers of cells thus reflecting specific interactions between the cells and the unique peptide sequence grafted on the bead [for a review, see (16)]. For instance a cyclic 8-mer peptide with the ability to attach a human ovarian adenocarcinoma cell line has been selected this way (17). Finally, other peptides such as the somatostatin analogs have been designed following a third method derived from direct structure-activity relationship studies made from the native full-length ligand to target neuroendocrine tumors expressing a high-density of receptors (5). Once a specific binding peptide has been identified for a given receptor, the peptide chemistry offers a full set of potential modifications aimed at improving its binding selectivity/affinity. The discovery of such short peptides paved the way to the development of radiolabeled-peptides (or analogs) as potential valuable tools for the detection and the treatment of cancer cells as reported in a recent review (12). Several selective receptor-targeting-peptides have emerged as potent radiopharmaceutical molecules upon their coupling to γ- or β-emitting radionuclides, in order to either visualize non-invasively receptor-expressing tumors or to eradicate receptor-expressing cells, respectively. Among these peptide receptors overexpressed on tumor cells, somatostatin, integrins, bombesin, cholecystokinin (CCK) gastrin, substance P, vasointestinal peptide (VIP), and neuropeptide Y have been extensively studied from the native sequence to the evaluation of analogs with better recognition properties (12).

Meanwhile several receptors overexpressed on various cancer cells have been characterized (AMHR, Axl, the HER series etc.) [for recent review, see (18)] but no peptide sequence with interesting binding affinities for these receptors has been so far identified. We can thus predict in the next years the identification of new short peptides (ideally less than 10 amino acids) as potential targeting tools for a large family of receptors differentially expressed by tumor cells.

## Improvement of the Binding Properties of the Peptides

Once the native sequence of a binding peptide has been determined, several criteria will be worth considering for reaching the highest tumor targeting properties. These include the design of more active or stable analogs differing from the original molecule by changes in their primary structure at well-defined position, the cyclization, or the multimerization of the targeting-peptide, driven by the extensive determination of the *in vivo* pharmacokinetics parameters including the accurate measurement of the biodistribution. The type of grafting of the radionuclide to the targeting-peptide, either directly or *via* a chelator, and the nature of the chelator itself have been also reported to be an important issue. Altogether it appears that the efficacy of the peptide-targeting unit relies on the individual influence of each structural parameter.

A significant number of peptide sequences with high binding capacity and selectivity for a specific cell receptor have been already identified [for a recent review: (12)]. These sequences usually contain between 3 and 10 amino acids. Based on the 20 amino acids naturally incorporated in the proteins, the chance to get a unique sequence is comprised between 20^3^ and 20^10^ which corresponds to one chance out of 8000 to more than 10,000 billions of possibilities. It sounds thus statistically reasonable to define a unique peptide sequence with a peculiar specificity for most of the receptors. Moreover, some amino acids could be replaced by analogs and therefore could increase the level of specificity for a given subtype of receptors.

Many examples from the literature highlighted that a circular peptide usually showed an overall increased affinity compared to its corresponding linear form. This was the case for renin analogs (19), but also for the RGD-derived-peptides those binding to the integrin receptors family has been extensively studied (20). Therefore, cyclization of these short peptide ligands has been previously proposed to induce structural constraints generally more favorable for interacting with their target protein. Aside the critical structural constraints associated with the cyclization to mediate a better rigidity and consequently an increased affinity for the targeted receptors, the cyclization also significantly protects the targeting-peptide from anticipated exoproteolytic cleavage occurring in biological fluids (21). Intramolecular cyclizations can be ensured either by the oxidation of two cysteine residues inserted at both ends of the primary sequence of the interacting peptide or by the formation of an amide bond between the N- and the C-terminal ends or, alternatively *via* other covalent closures depending on the type of chemical functions integrated on the linear peptide structure. Along this line, we recently synthesized various RGD-derived-peptides closed intramolecularly with an urea bond between the ϵ-amine of a lysine residue and the α-amino group of the peptide (22). Altogether a better stability and a higher affinity of the targeting-peptide for its target should ultimately significantly reduce the doses to be injected without impairing the diagnostic or therapeutic accumulation at the tumor sites.

**Figure 1:**
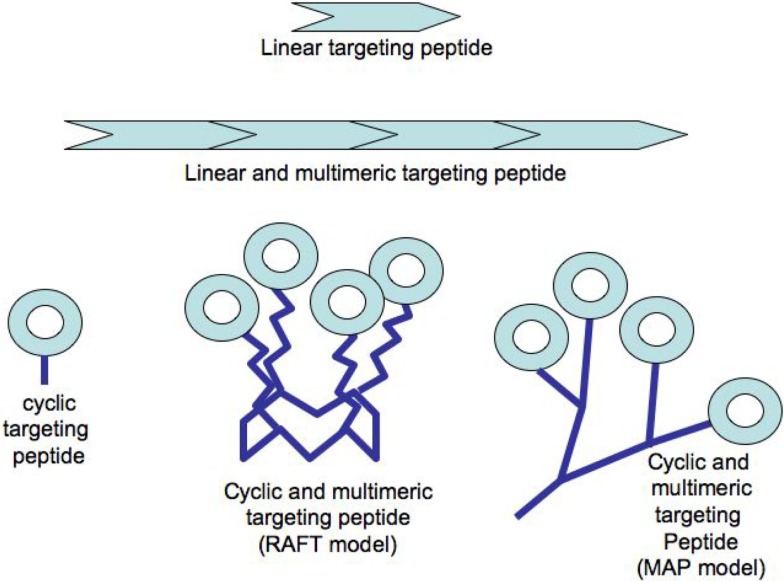
**Examples of peptide constructs used in radiotherapy or imaging**.

The multimerization of a binding molecule has also been considered as an important factor to improve the overall avidity toward a targeted receptor [for a review: (11)] and several recent studies confirmed this (10, 23, 24). The binding of a ligand is known to be a dynamic process with continuous binding and unbinding to its receptor. Thus, the apparent increase of the avidity of multimeric structures is explained by the unbinding of a ligand from a receptor site can be more rapidly replaced by the binding of another ligand covalently coupled to the first binding unit (25). Peptide chemistry offers a plethora of possibilities to build multimeric targeting-structures either by the successive duplication of a targeting sequence over a linear peptide or by grafting several cyclic peptides onto a multi-headed scaffold (see Figure 1). First, the simple duplication of a linear targeting sequence showed an improvement of the targeted receptor selectivity and affinity compared to the single sequence (26, 27). However such linear structure required the synthesis of a long peptide when considering ideally the repeat of two to four 7–8 amino acids long peptides. Moreover the exposure of the different repeats to the targeted receptor is probably not optimized under this linear form as already highlighted above (19, 20).

Significant improvement of the targeting capacity of such cyclic peptides has been ensured by the multimerization of the targeting-peptides using various scaffold units. Most of these scaffolds have been made from a peptide-based backbone (see Figure 1). These include the multiple-antigen presenting peptide (MAP) prepared with a lysine core initially designed for increasing the immunogenic response of short peptides upon injection in a host animal (28), or a peptide ring known as “regioselectively addressable functionalized template” (RAFT) and harboring four anchoring functions each allowing the grafting of one targeting-peptide (23). Both technologies were designed with at least one additional grafting function to attach directly or indirectly a payload moiety dedicated either to Peptide Receptor Imaging (PRI) or to Peptide Receptor Radionuclide Therapy (PRTT) as stated below. The clinical development of such peptide-targeting structures can be reasonably reached with all the criteria required for clinical use in peptide receptor-mediated radio-imaging or -therapy since as reported above, several peptide-derived molecules have been already provided to the clinic. The industrial synthesis of peptides is nowadays a routine process since pharmaceutical industries developed about 60 approved peptidic drugs for various pathologies with an annual sale of approximately US$ 13 billions in 2010. Quantitatively, there is almost a no-limit production scale since the production of several tons of Fuzeon^®^, an HIV antifusion 36-mers peptide (29).

We already mentioned the advantages of the relative small size of these peptide structures to more efficiently reach tumor cells deep within tissues. But their small size is also directly associated with a rapid clearance from the blood stream as usually observed for molecules less than 20 kDa. This fast elimination from the blood stream has been often presented as an advantage because of the reduction of the exposure time of healthy organs to the radionuclide attached on the peptide-targeting unit. However, the massive clearance through the kidneys and the liver machinery exposed more particularly these organs to the radionuclide radiation. For instance, renal irradiation is significant because of the prolonged residence time due to the reabsorption of the radiopeptide from the proximal tubuli and its resulting retention in the interstitium, predominantly in the inner cortical zone. Several strategies have been investigated to limit the rapid elimination from the blood stream and consequently, to reduce the renal residence time in kidneys. Along this line the combined injection of a radiolabeled targeting-peptide with a succinylated gelatin reduced its renal retention by almost 50% (30, 31). Amifostine, a small cationic molecule, has also been used to similarly lower renal retention not only for peptides (32), but also to limit the cumulative renal toxicity of cisplatin (33). Alternatively, co-infusion of targeting-peptides with free lysine or arginine cationic amino acids decreased significantly the renal tubular reabsorption of radiolabeled-peptides (34–36). More interestingly, a four fold reduction of the renal uptake has been recorded when a cationic lysine amino acid instead of a anionic glutamate amino acid was directly incorporated in the octreotide, a cyclic peptide used in clinic to target the somatostatin receptor (37). Altogether, these results highlight that several alternative strategies are already available to bypass or at least to limit the problems related to the *in vivo* use of small targeting-peptides.

Other different behaviors have been observed in the fate of two series of very related analogs, namely Demobesin-3 and -4 on one hand, and Demobesin-5 and -6 on the other hand (9). Demobesin-3 and -4 contained five more amino acids than Demobesin-5 and -6 made of a eight amino acids core. Despite an apparent minimal modification of the physico-properties of these peptides, it was shown that Demobesin-3 and -4 were cleared predominantly *via* the kidneys into the urine while Demobesin-5 and -6 showed a higher hepatobiliary excretion (9). Another discrepancy is the way these two series of peptide are differently taken up by the intestinal barrier (9).

In line with the reduction of the passive elimination of targeting-peptides through the kidney and/or the liver, different options have been proposed to modify the intrinsic peptide structure. For instance, due to a noticeable hydrophobic character most of the radiolabeled bombesin derivatives showed a high accumulation in the liver and a strong hepatobiliary excretion. This accumulation makes the use of such peptides unfavorable for the imaging of abdominal lesions, and also will highjack a substantial amount of a therapeutic peptides from the targeted tumor tissues. In order to tackle this problem, hydrophilic carbohydrated linker moieties were introduced into the bombesin analogs (38). As expected, this modification significantly improved the tumor-to-background ratios. PEG has been also proposed to increase the hydrophilicity of circulating moieties such as peptides (39) or much bigger structures such as liposomes or nanoparticles [for a recent review, see (40)]. Moreover PEG reduces the sensitivity of peptides to proteolysis because of a steric shielding of the peptide (41). Since PEGylation enlarges very importantly the molecular weight of the peptide, it also reduces significantly the kidneys ultrafiltration, particularly if the PEG size exceeds 30 kDa (42). On the other hand, mini-PEG polymers made with eight-carbon chains prolonged the metabolic half-life of a targeting-peptide leading to higher target-to-background ratios and improved *in vivo* PET imaging of inflammation (43). PEG modification of large targeting structures appears to automatically trigger an enhanced permeability and retention (EPR) effect in the tumor (44) leading to an overall increase of the therapeutic effect. However, the EPR effect was found to be effective only for molecules with a molecular size >45 kDa [for a review (45)]. Therefore, this is not applicable for short targeting-peptides because it is likely that their PEGylation could alter the binding capacity and/or selectivity. PEGylation has been recently developed to shield a small targeting-peptide (RGD) and to reduce the transport from the tumor interstitium to the vascular compartment (46). However, it appears that to be fully effective, this strategy, named Diffusion Molecular Retention (DMR), required a peritumoral injection of the complex.

The way these peptides are differently delivered in or eliminated from the blood stream is likely the direct consequence of their respective intrinsic physico-chemical properties. The cell internalization of a targeting-peptide and therefore its biological activity could be also directly affected depending on small changes within its primary structure. As an example, the single presence of a positive charge at the N-terminus of a peptide ligand induced a faster and higher cellular internalization of bombesin analogs in gastrin releasing-peptide receptor-expressing cells (47). Last but not least, major differences could be eventually observed in the metabolic stability of peptide-targeting units. Along this line, the metabolic degradation of the different Demobesin analogs has been shown to be slow in mouse plasma *in vitro*. However upon intravenous injection, their behavior appeared very different since Demobesin-3 and -4 were both recovered as a very hydrophilic metabolite whereas the shorter forms (Demobesin-5 and -6) generated two major metabolic species (9). For all the analogs, the cleavage was not related to the direct *in vivo* breakdown of the metal chelator *per se* but rather to the cleavage of amide bonds either directly within the peptide or between the metal chelator and the peptide.

In conclusion, since limited changes in the targeting-peptide could modify the *in vivo* pharmacokinetics behavior of two very related peptides (diffusion, stability, cell uptake, and elimination), it is worth considering that every single modification at different levels of the targeting unit could significantly modify the overall efficacy of the targeting-peptide. This implies a full and very rigorous re-evaluation of the peptide-targeting behavior following a marginal modification of the targeting unit but also paves the way to more extensive structure-activity relationship studies upon the replacement of amino acids by various peptidomimetics of different characters to further optimize the efficacy of each peptide receptor-mediated therapy.

Another issue raised about peptide receptor-mediated targeting relies on the use of antagonists or agonists of the targeted receptor. It has been shown both *in vitro* and *in vivo* that receptor antagonists might be preferable to agonists (48). It has first to be recalled that an agonist drug binds to the same site than the endogenous ligand and triggers the same expected biological effect. Conversely the shape of an antagonist is close enough to bind to the binding site onto the receptor, but it does not produce any subsequent effect such as the internalization of the receptor. For a therapeutic purpose, it sounds preferable however to induce the rapid cellular internalization of the radiolabeled targeting-peptide to avoid its displacement due to the binding of the endogenous ligand. Thus, the quicker the receptor internalizes the more the cell will be loaded with the radionuclide. However, we reported that the plasma membrane was a more sensitive target than cytoplasm to dense ionization produced by Auger electrons when using either a non-internalizing or an internalizing ^125^I-labeled antibody (49–51). This higher toxicity will have to be further investigated depending on the categories of radionuclides (for instance Auger emitters versus alpha emitters). If this deleterious activity at the plasma membrane were confirmed, the cellular internalization of a radionuclide coupled to an agonist molecule would be optional.

The much lower antigenicity of short peptides compared to larger molecular structures is also presented as an advantage to support the development of peptide-based targeting units since it cannot be excluded that in a therapeutic process, several injections of the peptide-based drug over a long period could be necessary to fully destroy the tumor cells.

## Choice of the Chelator and the Radionuclide

In addition to the peptide that serves to carry the radionuclide to the tumor target, other parameters must also be considered when designing optimal radiotherapeutic peptide tools for *in vivo* development. These include the radionuclide and the chelator bound on the targeting-peptide, but also the location at which the chelator has been grafted on the targeting-peptide because the difference in the regional charge distribution may differentially alter receptor-binding, non-specific organ uptake, and hence the *in vivo* biodistribution characteristics (52, 53). The radionuclide is selected based on its physical, chemical, and biological properties and should ideally be routinely available, easy to couple to the chelator, harbor short range, high energy, and abundant particle emission, a stable daughter product, and an appropriate physical half-life to selectively eliminate the targeted neoplastic tissue while sparing normal ones. The optimal chelating agent should fulfill the following criteria: its addition should not interfere with the specificity or with the binding of the peptide to its target receptor, neither alter its rate of catabolism or patterns of tissue distribution. Moreover since it is the most common cause of failure for PRI or PRRT, the chelator should tightly hold the radiometals to avoid their premature elution *in vivo* and the risk to be delivered to normal tissues where they cause toxicity (54). Finally during its elimination, the chelator should not slow down the clearance of the radiometal following the complete catabolism of the targeting-peptide.

The therapeutic radionuclides used for labeling peptides are yttrium (^90^Y), lutetium (^177^Lu), rhenium (^188^Re, ^186^Re), copper (^67^Cu, ^64^Cu), and indium (^111^In). They emit low linear energy transfer (LET) radiation of 0.2 keV/μm in the form of beta-particles, internal conversion electrons, as well as gamma-rays or X-rays that makes them suitable for imaging, such as ^64^Cu and ^90^Y for Positron Emission Tomography, ^67^Cu, ^177^Lu, ^111^In, and ^188^Re for Single-Photon Emission Computed Tomography, and ^186^Re for planar scintigraphy (55–58). When low LET radiation interacts with a cell, it produces sparse ionization and individual DNA lesions easily repairable by the cellular machinery. Consequently, low LET radiation mainly causes sublethal damage, but higher lethality rate can be reached either by increasing the absorbed dose or by fractionating the therapy. To concentrate the highest absorbed dose to the tumor, it is important to choose a radionuclide for which the half-life matches as much as possible the pharmacokinetics of the targeting-peptide. Other therapeutic radionuclides with higher LET radiation (50–230 keV/μm) emitters are available. These include bismuth (^213^Bi, ^212^Bi) and astate (^211^At), as well as lead (^212^Pb) and actinium (^225^Ac) since they generate ^212^Bi and ^213^Bi as daughter components, respectively. These radioisotopes emit alpha particles that can produce DNA double-strand breaks even with a single radiation hit and generate clusters of DNA damage in a small volume which are poorly repairable. Thus, these emitters induce higher cytotoxicity independently of the dose rate (59).

Yttrium, lutetium, rhenium, copper, lead, actinium as well as bismuth are metal radionuclides. Their conjugation to the targeting-peptides requires a chelating agent, an organic ligand named chelator (Table 1) (whereas iodine or fluorine labeling is based on substitution or addition directly on the targeting-peptide). The chelating agent must be bifunctional because it has to coordinate in one hand the radiometal and in the other hand to covalently link the peptide moiety through a functional group (60). The choice of the bifunctional chelator (BFC) is defined by the nature and oxidation state of the radiometal that determine the coordination chemistry thus forming the chelate (61). The paramount characteristic of a BFC lies in its high thermodynamic stability and kinetic inertness to avoid the release of ionic radionuclide under the physiological conditions (61). The decomposition of the BFC can be the result of the formation of free radicals generated by radiolysis, the chemical modification of the BFC, and subsequently, the production of free metal ions leading to radiotoxicity. Another point is stereoisomerism of the metal chelate leading to different spatial orientation of the compound that might affect lipophilicity and, as described for the peptide modification, changes in the biodistribution pattern. However biodistribution is directly dependent on blood clearance closely related with the hydrophilicity of BFC which influence renal excretion of the conjugate (61). All these points will be evaluated to choose the best BFC required.

**Table 1:** **Chelator families and their representative for metal complexation**.

Natural chelator	Desferrioxamine	
		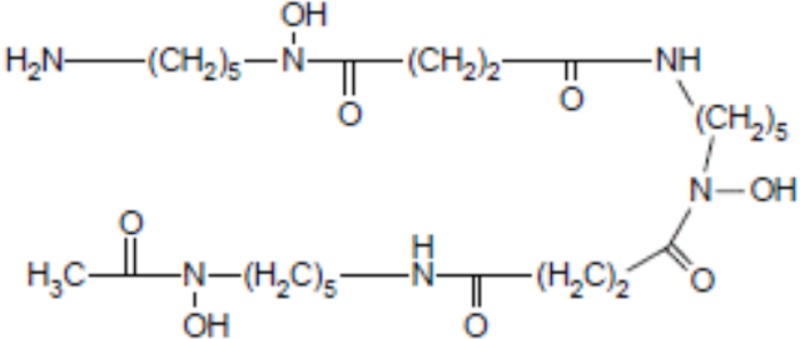
Acyclic polyaminocarboxylate ligands	EDTA, Ethylenediaminetetraacetic acid	
		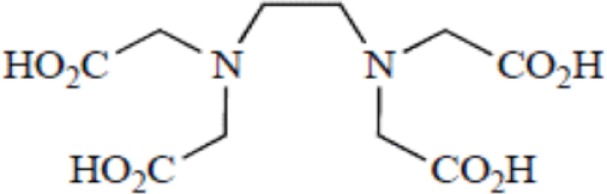
	DTPA, Diethylenetriaminepentaacetic acid	
		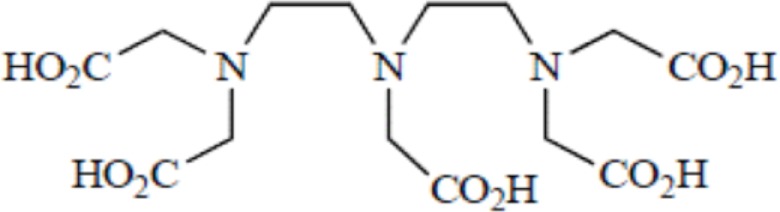
Cyclic polyamines	Cyclam, 1,4,8,11-tetraazacyclotetradecane	
		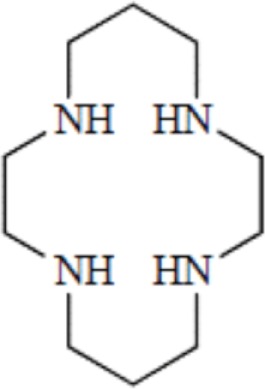
	Cyclen, 1,4,7,10-tetraazacyclododecane	
		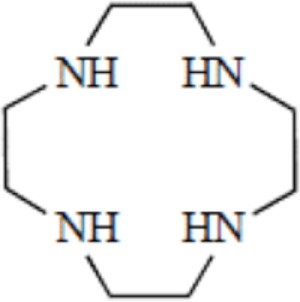
Cyclic polyaminocarboxylates and their derivatives	DOTA, 1,4,7,10-tetraazacyclododecane-1,4,7,10-tetraacetic acid	
		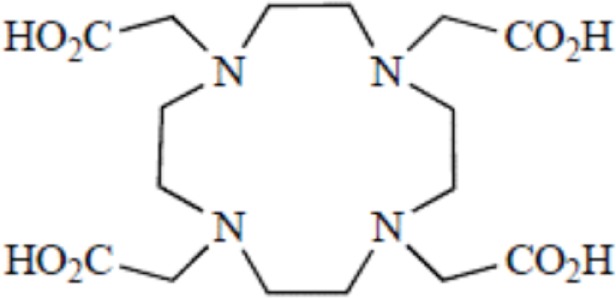
	TETA, 1,4,8,11-tetraazacyclotetradecane-1,4,8,11-tetraacetic acid	
		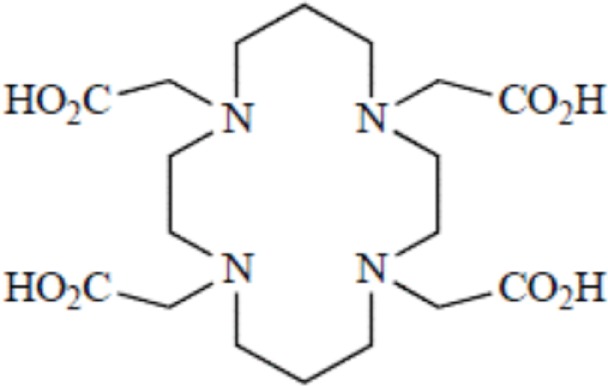
	NOTA, 1,4,7-triazacyclononane-1,4,7-triacetic acid	
		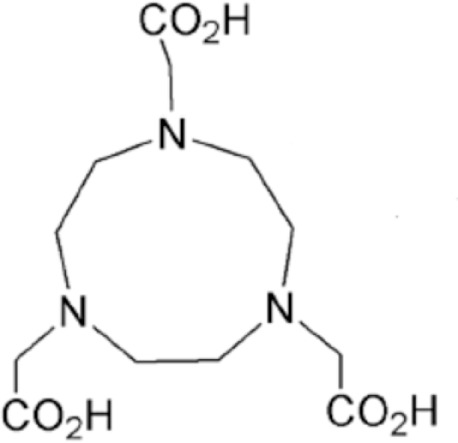
Amines with rigid backbones	Tachpyr, *cis,cis*-1,3,5-triaminocyclohexane	
		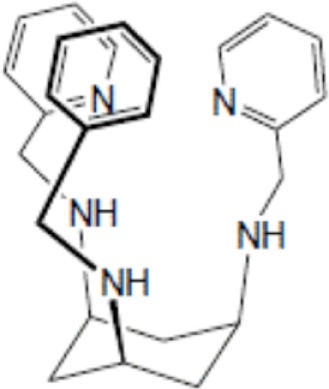
Cross-bridged cyclic polyaminocarboxylates	CB-TE2A, 1,4,8,11-tetraazabicyclo[6.6.2]hexadecane	
		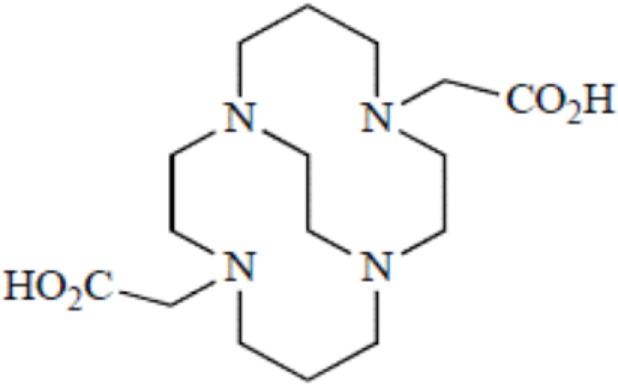
Bicyclic hexaamine	Sar, sarcophagine	
		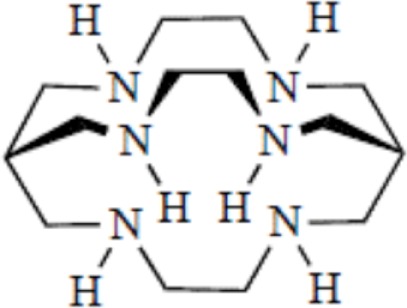

Cyclic polyamines are also named polyaza macrocycles; note that the sarcophagine (bicyclic hexamines) belongs to this family. Adapted from Wadas et al. (60) and Brechbiel et al. (62).

### Optimal coordination

Several criteria have to be considered to optimize the coordination of the metal and therefore the stability of the metal on the chelator. These include the charge, the match between the ionic radius of the radionuclide and the cavity size of the chelator, the appropriate denticity of the chelator acting as a set of firm pincers binding of certain metal ions (associated to the number of donor binding groups) with the appropriate chemical characteristics. The rate of formation and dissociation of the metal complex has also to be considered (62). Taking into account all these parameters will ensure the strongest stability and will limit the dissociation, leading to a high thermodynamic stability and kinetic inertness.

### Thermodynamic stability and kinetic inertness

Apart from the natural desferrioxamine, one of the earliest BFC used (62), the polyaminocarboylate ligands, also known as acyclic polyamine carboxylate ligands, through bifunctional EDTA have been first used to chelate various radionuclides. EDTA is an open-chain ligand and donates its six lone pairs of electrons to coordinate metal cations (named hexadentate ligand). A derivative of EDTA, DTPA, is an eight-coordinating complexing agent forming complexes of 2–4 orders of magnitude higher than EDTA thus allowing the coordination of larger metal ions such as lanthanides with coordination numbers of 8 or 9 (63). Indeed the larger number of ring closures around the metal atom, the more stable the complex.

Another wide family of metal chelators is the tetraazamacrocyclic ligands group. These macrocycles tightly encapsulate metal ions. DOTA, a dodecane backbone derivative, is the most extensively used representative of these macrocycles. The “tetraaza cage class” of complexing agents offers a good thermodynamic stability (i.e., stability constant) of the divalent metals complex as Cu^2+^ for copper isotopes (typically ^64^Cu and ^67^Cu) (64).

Although an apparent strong complexation *in vitro*, ^64^Cu-TETA-octreotide has been reported to partly loose the radionuclide *in vivo* despite the tetradecane basis of TETA (65) (see toxicity paragraph). This phenomenon has been also described for linear or macrocyclic BFCs shown to be unstable in human serum over long periods of 2 or 3 days (66). However, macrocyclic or macrobicyclic chelators are generally good choices for encapsulating this category of radioisotopes. These ligands utilize both the macrocyclic and chelate effects to enhance stability (60) probably because when one of the chelating nitrogen atom dissociates from the metal center, the overall topological constraints of the cage still maintain the metal inside and ensure its facile re-coordination to an other nitrogen (67).

The moderate *in vivo* stability of ^64^Cu-DOTA could increase the non-targeted organ radiation dosage and lower the tumor-to-non-tumor contrast compared to non-macrocyclic agents. ^64^Cu-labeled radiopharmaceuticals with improved stability have been reported (68). These include NOTA derivatives and CB-TE2A, a cross-bridged version of TETA where two of the acetate arms have been replaced by ethylene bridges between non-adjacent nitrogens. Such bridging has been shown to improve the stability of Cu-cyclam, probably because when one of the chelating nitrogen atoms of the cage dissociates from the metal center, the topological constraints induced by the ligand do not allow it to move very far away from the metal center, effectively ensuring its facile re-coordination (67). Cross-bridging led to an exceptional kinetic inertness in aqueous solution (69) and high resistance to acid catalyzed decomplexation experiments, better than other chelator such as sarcophagine, a hexaazamacrobicyclic chelator able to form fully encapsulated complexes with copper (60). Along these lines the sarcophagine (denoted as “Sar”) and its derivatives are well known to strongly bind copper and to generate highly stable complexes (70). The better stability of Hg(II)-Sar complexes over Hg(II)-DOTA, Hg(II)-TETA, or Hg(II)-cyclam has been thus confirmed (71).

Kinetic stability plays a more central role in biological stability of metal-complexes than thermodynamic stability *in vivo*. The thermodynamic stability is directly related to the energies involved in the metal complex formation. The kinetic stability provides direct insights into relative *in vivo* dissociation by describing the kinetics of dechelation corresponding to the reaction occurring *via* spontaneous dissociation of the chelate or the kinetic of transmetallation due to endogenous metals (72). Therefore the slower a reaction, the greater the kinetic stability. The effect of increasing macrocycle size from DOTA to TETA results in a small decrease in the thermodynamic stability for the relatively small Cu^2+^ ion diameter. The correctness of this relationship between thermodynamic and kinetic stability still needs to be demonstrated since the decrease in thermodynamic stability seemed inconsistent with the greater *in vivo* stability of ^64^Cu-TETA for instance (73). Thermodynamic stability and acid stability measurements for other radionuclide-chelator complexes did not accurately predict either the *in vivo* stability (56). Identically, in a series of six Cu(II) macrocyclic complexes, some of the most thermodynamically stable complexes appeared to be the least stable *in vivo*, which confirmed the kinetic stability as a crucial parameter to be considered (74). On the other hand, a class of cyclic polyamine ligands showing relatively high stability both *in vitro* and *in vivo* when complexed to Cu(II) are the methylenephosphonate pendant armed tetraazamacrocyclic ligands. However, the phosphonate function present on this chelator has been shown to be a natural bone-seeking agent (75, 76). Therefore, the use of this class of chelators has been very rapidly abandoned for obvious toxicological reasons.

Bifunctional chelators are thus essential components in the assembly of radiometal-labeled-peptides since they play a critical role in the *in vivo* stability of the radioconjugate and, thus their therapeutic effectiveness (54) despite the lack of a clear understanding of the relationship between thermodynamic and kinetic stability. However, it has been empirically shown that macrocyclic ligands were usually the most kinetically stable chelate-complexes in human serum. To gage the efficiency of new chelate-complexes, DOTA remains considered as the gold standard of kinetic stability and is often used as a reference (77). For acyclic ligands complexed with a series of lanthanide metals, it has been predicted that flexibility was critical for stability (78). As an example, it was suggested that inflexible conformations of the DTPA chelator complexed with various lanthanide metals showed a decreased stability correlated with the decrease of the metal atomic radii. But again this theory has been reconsidered since other stability studies led to less clear results despite the use of identical lanthanide metal complexed with a more rigid DTPA chelate complex (77). Thus, prediction of kinetic stability with acyclic ligands on the basis of metal radius size also appears more complicated than expected.

Several new ligand systems, including those based upon the *cis*, *cis*-1,3,5-triaminocyclohexane scaffold, the sarcophagine ligands, and the cross-bridged tetraazamacrocycles have been developed to complex ^64^Cu more stably. Inside the *cis*, *cis*-1,3,5-triaminocyclohexane family the ^67^Cu-tachpyr complex exhibit a higher stability in human serum than the ^67^Cu-[tachpyr-(6-Me)] (79). DiBartolo et al. investigated a family of Sar derivatives, for which complexation was 100% complete within several minutes at 25°C over the entire pH range (80).

### Toxicity, transmetallation, and transchelation phenomenons

Because of the geometrical constraints of macrocyclic BFCs, the spontaneous dissociation of the metal from this type of chelator should be limited thanks to the potential re-coordination of the metal ion. Metal ions naturally present in blood and other body fluids can also displace metal radionuclide ions from their complex (the so-called “transmetallation” phenomenon) (72). As seen above, the loss of the metal can occur thus impeding its localization to the target sites. Consequently, this decomplexation can create critical problems for imaging because of an important increase of the background signal but more importantly, there is a major toxicity concern due to the long-term accumulation of lanthanide elements in bones, with the risks of deleterious bone marrow irradiation (56). The decomplexation of copper can lead to its sequestration in metallothionein in hepatocytes and then its exportation to other organs following re-circulation in blood (64). This has been particularly noticeable with the important retention of ^64^Cu activity in blood, a poor liver clearance, and an increasing bone marrow uptake leading to a significant drop of the number of white blood cells despite weak signs of overall toxicity in rats (65).

Another factor affects stability and refers to the exchange of ligands: the transligation, or transchelation phenomenon. It is observed when the used metallic radionuclide is scavenged by a metalloenzyme that can naturally complex a metal of similar or very close ionic radius. The transchelation of ^64^Cu from DOTA to superoxide dismutase (SOD) in the liver has been for example observed (73). Therefore, a ^64^Cu-DOTA therapy is indeed difficult to encourage owing to the persistent presence of the final radiometal metabolite (^64^Cu-DOTA-lysine) within the liver (70). An identical transchelation has been also shown *in vivo* with a TETA-octreotide conjugated with a accumulation of ^64^Cu bound to SOD in rat liver (81). As illustrated with this example dealing, there is a great need to address these transchelation concerns and to design chelators forming more stable complexes with copper.

### Clearance

The blood clearance of radiolabeled-chelators also appears to be very related to the structure of the used compounds. As an example, Boswell and coworkers demonstrated better blood clearance properties through the liver and kidneys for a cross-bridged BFC than for an azamacrocycle BFC analog (73). Apart from the cross-bridged structure, the charge of the BFC could induce a significant effect on the kidney and liver clearance since it has been shown that CPTA or PBCA with a net charge of +1 showed much greater accumulation in the kidney and the liver and, consequently, a slower clearance than the BFC with a net formal charge of −2 (82). In addition to the difference in the net charge of these chelators, there is also a major difference in the physico-chemical characters since CPTA and PBCA are much more lipophilic and positively charged compared to BAT and TETA. The respective influence of the structure, the charge, and the lipophilic characters of the chelator components, as well as the targeting-peptides as developed above, underlying the blood clearance is not currently fully understood, nor can be anticipated the consequence on the blood clearance following a despite seemingly small chemical modifications of one component of the targeting structure.

### Labeling conditions

Complexation procedures of radionuclides to chelators have also been the subject of several investigations to improve the yield of labeling while limiting the denaturation of the targeting unit to be functionalized. Such denaturation problems are rather limited with targeting-peptides because their spatial structure is usually more stable due to their short size or highly constraint structures. As a matter of fact, the DOTA radiochemistry requires for instance heating – up to 95°C for ^68^Ga labeling – to obtain adequate yields and specific activities (83, 84). However, NOTA was radiolabeled efficiently at much lower temperatures (40°C) (85) or at ambient temperature (86). The cross-bridged ligand CB-TE2A also requires high complexation temperature (95°C to label ^64^Cu with a high radiochemical purity). Altogether these data indicate that the more complex the chelator is, the higher temperature should be provided to complete the loading of the chelator with the corresponding radionuclide. Another important issue is obviously the stoichiometry ratios between the macrocyclic chelator and the metal since ratio of 1:2–1:3 are often required when complexing macrocyclic chelator whereas 1:1 ratio was sufficient for acyclic ligands. The chelation is also time-dependent depending on the macrocyclic or linear structure of the chelator (77).

These differences in the preparation conditions (temperature, stoichiometry, kinetics) likely reflect the increasing difficulty for the radionuclide to get access into the chelator with a higher degree of complexity.

## Conjugation of the Peptide with the BFC Using Heterolinkers

**Figure 2:**
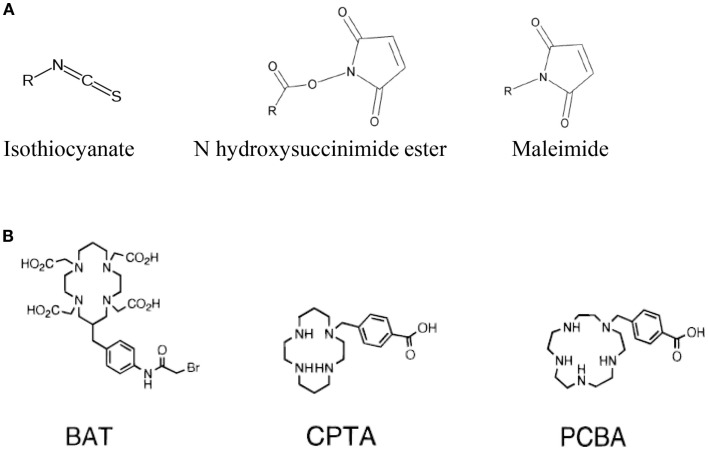
**Main functional groups used to covalently link the peptide moiety (A) and examples of BFCs (B)**. BAT is a derivative of TETA; CPTA is a derivative of cyclam, and PCBA is a derivative of cyclen (see Table 1). BAT is conjugated to peptide using the linking agent 2IT (2-iminothiolane) which forms a thioether bond between BAT and the peptide. CPTA and PCBA are conjugated to peptide *via* an amide linkage. Adapted from Rogers et al. (82).

The choice of the bifunctional linker to couple the targeting-peptide with the radionuclide-chelator is closely related to the chemistry encountered when performing the derivatization or conjugation of proteins, peptides, nucleic acids, or the functionalization of nanoparticles, liposomes, biological surfaces, etc. Indeed it depends on the type of functional group that is present on both the chelator and the targeting-peptide. Isothiocyanate (NCS), *N*-hydroxysuccinimide-esters (NHS-esters) or maleimide group are more commonly used for the covalent coupling of primary amines or thiols (see Figure 2) (70). Indeed, as observed with this type of molecule conjugation using these heterolinkers, there is a risk of competitive hydrolysis mainly with the NCS or NHS moieties that could indeed reduce conjugation efficiency. These secondary reactions could indeed be amplified particularly if high temperatures are required during the radionuclides loading on the chelator. Another major concern to consider is the possible interference of the conjugation of the chelator-radionuclide with the optimal properties of the targeting-peptide binding to its receptor since the conjugation site can be located at or near the active site (70). This is more the case for radioimmunotherapy upon conjugation of a chelator on a mAb than on a targeting-peptides because peptide chemistry offers a large panel of functionalization at various well-defined sites within the primary sequence to avoid steric hindrance.

There are a large numbers of heterolinkers available on the market from several suppliers. Some of them are also design to offer various characteristics depending on the needs. These include different lengths to get variable spacing between the coupled molecules, or different chemical natures to fit with the experimental requirements. Indeed, this offers a new site for modifying the biological behavior of the targeting unit by changing the physico-chemical character of this heterolinker moiety as recently proposed with the integration of mini-PEG spacering between a targeting-peptide and a 68Ga loaded DOTA chelator (43). More generally, peptide-targeting-BFCs loaded with a metal radionuclide can also be modified with pharmacokinetic modifying linkers (PKM) of different natures (cationic, anionic, neutral, or metabolically cleavable.). The linker can be a simple hydrocarbon chain to increase the lipophilicity, a peptide sequence to improve hydrophilicity, and renal clearance, or a PEG linker to slow extraction by hepatocytes (61).

## Conclusion

In molecular terms, the difference between PRI and PRRT in a peptide receptor strategy is only limited by the type of radionuclide loaded on its chelator, this latter being itself covalently bound to the targeting-peptide. Since both PRI and PRRT strategies could be based on the same targeting-peptide, it offers the possibility to perform first a PRI with a given couple of chelator-radionuclide and to treat afterward the patient with the same targeting-peptide but now loaded with a more cytotoxic radionuclide to induce a PRRT response. This possibility could allow the accurate determination of the personalized biokinetics constants associated with the delivered targeting-peptide before the therapeutic phase using the same targeting-peptide but now loaded with a compatible radionuclide mounted on its respective chelator. When possible, the adjustment of the injected radiation dose and the prediction of the therapeutic efficacy on tumor lesions as well as effects on healthy organs can be better anticipated. After the radiotherapy phase, PRI can be once again used to assess the progression, the stability, or the relapse of the tumor under the same initial conditions previously recorded.

Moreover, thanks to the versatility to attach any chemical moiety onto a targeting-peptide, one can imagine the concomitant use of a drug- and a radionuclide-targeting-peptide to induce a more potent therapeutic effect. In line with this, the combination of peptide receptor-mediated radionuclide therapy using octreotate or octreotide somatostatin binding peptides with fractionated external beam radiotherapy has been recently evaluated in patients suffering of advanced symptomatic meningioma. All patients reported stabilization or improvement of tumor-associated symptoms, without any morphologic tumor progression (87).

The use of a combination of two (or more) radiolabeled targeting-peptides can lead to a number of advantages. The somatostatin receptor is the best example for illustrating this issue since tumor cells overexpressed concomitantly, but at different expression level, the somatostatin heterodimeric receptors (sst1-5). Each of these receptor dimers binds the somatostatin derived-peptides with different affinity (1). Therefore, the use of a cocktail of radionuclide labeled-peptides could increase in this case the overall concentration of the radioactivity at the tumor level following a cumulative effect of each peptide bound to its own cell-target. Second, the expression of these peptide receptors is likely heterogeneous, both spatially and temporarily. For instance, the expression rate of one of the receptors could decrease over the duration of the treatment and consequently, the use of several targeting-peptides could thus prevent the reduction of the therapeutic effect during the treatment. Altogether the use of several targeting-peptides at the same time could significantly improve the overall efficacy of the PRRT.

## Future Directions with Tumor Targeting-Peptides

Peptide chemistry offers an almost limitless panel of possibilities to synthesize peptide molecules aimed at targeting a tumor receptor expressed more exclusively on tumor cells. We previously mentioned the cyclization to stabilize the peptide integrity or to increase the peptide affinity, the multimerization to augment the avidity, the insertion of unnatural amino acids mainly to improve the peptide stability toward proteases but also in some cases to modify the binding specificities. The addition of biologically compatible components such as PEG could also alter the biokinetics characters, and consequently the bioavailability of the targeting unit. Indeed since all the recptors are different, there will probably not be a well-defined strategy to apply universally and it is likely that investigations will have to be made in each case. For some specific neoplasms, it might be possible to combine several characteristics from individual different peptides to impulse a synergistic effect toward the targeting unit. This has been recently initiated by the Ruoslahti’s group which used two peptides built in one single linear sequence (88). The described system is based on the use of an integrin binding peptide (sequence CRGDK) linked to a cryptic CendR peptide (R/KXXR/K-OH). It has been demonstrated that the first RGD peptide bound the alpha-v beta-3 integrin receptor and then, following a not-yet-identified mechanism, a proteolytic event occured thus exposing the CendR motif which became able to interact with NRP-1 receptor and to trigger an improved internalization process only in tumor cell targeted by the RGD peptide (88).

Several peptides have been identified as potential tools to target tumor cells and most are currently still under investigation (see Table 2). As previously discussed, it might be advantageous to target several receptors at the same time. This can be performed upon the co-injection of two different targeting units, but we can also imagine a targeting unit loaded with two (or more) different targeting-heads in order to bind a wider set of tumor cells expressing different receptors in different abundances. As previously described, the peptide synthesis methodology offers the possibility to use the same targeting head but loaded with different effective moieties, either dedicated to perform a PRI or a PRRT, depending on the type of radionuclide, or a chemotherapy upon the grafting of a cytotoxic agent. This offers the possibility to fight a tumor with different therapeutics weapons based on the same recognition mean.

**Table 2:** **Peptides identified as potential tools to target tumor cells**.

Target receptor	Sequence	Length	Reference
EphA2 (TK)	YSAYPDSVPMMS	12AA	Wang et al. (89)
CCK/Gastrin	qqqAYGWMDF	10AA	Chopra (90)
p32 receptor	CGNKRTRGC	9AA	Karmali et al. (91)
α5β1	PHSCNK	6AA	Dai et al. (92)
EGFR	FALGEA	6AA	Leung (93)
Tumor vessels	CGKRK	5AA	Yao et al. (94)
α2β1	DGEA	4AA	Huang et al. (95)
APN (CD13)	NGR	3AA	Chen et al. (96)
αvβ3	RGD	3AA	Nahrwold et al. (97)

The use of targeting-peptide is particularly appropriate for some urogenitary cancer since a direct intraperitoneal injection of few milligrams of peptide could allow global targeting-peptide concentration in the micromolar range which should an optimal concentration to obtain good cellular binding of targeting-peptide to cell receptor.

## Conflict of Interest Statement

The authors declare that the research was conducted in the absence of any commercial or financial relationships that could be construed as a potential conflict of interest.

## Abbreviations

BAT, 6-[*p*-(bromoacetamido)benzyl]-1,4,8,11-tetraazacyclotetradecane-1,4,8,11-tetraacetic acid; CB-TE2A, 1,4,8,11-tetraazabicyclo[6.6.2]hexadecane; CPTA, 4-[(1,4,8,11-tetraazacyclotetradec-1-yl)methyl]benzoic acid; DOTA, 1,4,7,10-tetraazacyclododecane-1,4,7,10-tetraacetic acid; DTPA, diethylenetriaminepentaacetic acid; EDTA, ethylenediaminetetraacetic acid; NOTA, 1,4,7-triazacyclononane- 1,4,7-triacetic acid; PBCA, 1-[(1,4,7,10,13-pentaazacyclopentadecyl-1-yl)methyl]benzoicacid; TETA, 1,4,8,11-tetraazacyclotetradecane-1,4,8,11-tetraacetic acid.
